# Genomic Insights and Inactivation Strategies for *Lactiplantibacillus plantarum* Postbiotics Production

**DOI:** 10.3390/foods15122148

**Published:** 2026-06-14

**Authors:** Mia Radović, Tomislava Grgić, Martina Banić, Katarina Butorac, Andreja Leboš Pavunc, Jagoda Šušković, Jasna Novak, Blaženka Kos

**Affiliations:** Department of Biochemical Engineering, University of Zagreb Faculty of Food Technology and Biotechnology, Pierottijeva 6, 10000 Zagreb, Croatia; mradovic@pbf.hr (M.R.); tomislavagrgic5@gmail.com (T.G.); martina.banic@pbf.unizg.hr (M.B.); katarina.butorac@pbf.unizg.hr (K.B.); andreja.lebos.pavunc@pbf.unizg.hr (A.L.P.); jagoda.suskovic@pbf.unizg.hr (J.Š.); blazenka.kos@pbf.unizg.hr (B.K.)

**Keywords:** cell inactivation strategies, genomic characterization, lactic acid bacteria, *Lactiplantibacillus*, probiotics, postbiotics, sequencing, strain screening

## Abstract

Probiotic lactic acid bacteria are widely recognized for their health-promoting effects. However, the use of live microorganisms may pose safety concerns and stability limitations. Consequently, postbiotics, defined as inactivated microbial cells and/or their components, have emerged as a promising alternative. This study integrates genome-guided evaluation of probiotic potential, experimental validation of in silico predictions and process optimization for the production of inactivated *Lactiplantibacillus plantarum* DM1 and KK1 cells as postbiotics. Genome mining identified genes and gene clusters associated with metabolic versatility, antimicrobial activity, gastrointestinal stress tolerance, adhesion and prebiotic substrate utilization. Building on these findings, to generate postbiotics, the efficiency of thermal, enzymatic, mechanical and radiation-based inactivation methods was evaluated in bacterial suspensions prepared in three dairy by-product matrices: milk permeate, sweet whey and sour whey. Complete inactivation of both strain cells was achieved by thermal treatment (3 min pasteurization), *γ*-irradiation (3 kGy), and combined lysozyme–pasteurization treatment, whereas other treatments showed partial and matrix-dependent effects. Matrix composition significantly influenced treatment efficacy, suggesting a protective role of food components used. These findings highlight the importance of combining genome mining for potential probiotic strain characterization with robust, matrix-adapted inactivation strategies for the development of stable postbiotic formulations.

## 1. Introduction

Most probiotic microorganisms belong to Gram-positive, non-pathogenic Lactic Acid Bacteria (LAB), which naturally inhabit diverse ecological niches, including plants, milk, and the gastrointestinal tract [1,2]. These bacteria ferment carbohydrates and produce substantial amounts of lactic acid, associated with beneficial health effects [3]. However, despite these well-documented health-promoting properties, the use of live probiotic microorganisms is not without risks. Although many LAB strains have Generally Recognized as Safe (GRAS) status and are widely considered safe, adverse effects have been reported in vulnerable populations, including gastrointestinal disturbances, bloodstream infections, intestinal dysbiosis, and horizontal gene transfer, particularly of antibiotic resistance genes [4,5]. Of particular concern is the potential spread of antimicrobial resistance, which represents a significant public health threat [4].

Owing to these limitations, increasing attention has been directed toward the investigation of non-viable (inactivated) forms of probiotic bacteria. It has been demonstrated that many beneficial immunomodulatory and other biological functions do not necessarily depend on bacterial viability or replication capacity, but rather on structural components and metabolites of the cells [6]. This concept has led to the emergence of the term *postbiotics*, defined as non-viable microbial cells and/or their components that confer health benefits to the host [7]. Other related terms have also been used in scientific literature, such as *paraprobiotics* for non-viable probiotic cells, and *postbiotics* for microbial by-products [6]. However, these earlier distinctions have largely been superseded by the current consensus definition of postbiotics [7]. In contrast to live probiotics, postbiotics offer several advantages, including improved safety, enhanced storage stability, and greater resistance to environmental fluctuations, making them attractive for applications in the food and pharmaceutical industries [8]. So far, various bacterial inactivation methods have been described, including thermal treatment [9], lysozyme (LYZ) treatment [10], sonication (ultrasound, US) [11], and combinations thereof [10]. The primary objective of these approaches is to halt bacterial replication while preserving surface structures and immunomodulatory potential. In this context, *γ*-irradiation has emerged as a particularly promising inactivation strategy. It enables the generation of metabolically active but non-replicative cells, bacteria that retain cellular structure and partial functional activity. Compared with thermal inactivation, *γ*-irradiation has been shown to better preserve surface antigens and cellular components. Several studies have even reported superior biological effects of such postbiotics relative to heat-inactivated counterparts in disease models. Evidence also suggests that different inactivation methods may differentially affect the retention of beneficial properties across bacterial strains, further highlighting the importance of selecting appropriate inactivation strategies [12].

Among LAB, *Lactiplantibacillus plantarum* has attracted attention due to its remarkable ecological versatility and long history of safe use in fermented foods [13]. Numerous *Lp. plantarum* strains have demonstrated probiotic potential, including the ability to tolerate gastrointestinal conditions, adhere to intestinal epithelial cells, modulate immune responses and produce bioactive compounds [14]. The availability of whole-genome sequencing (WGS) data has facilitated the identification of genetic determinants associated with probiotic functionality and safety, making *Lp. plantarum* an attractive candidate for postbiotic development [15]. Accordingly, comprehensive evaluation of probiotic candidates, including the identification of genes associated with stress tolerance, adhesion, bacteriocin and exopolysaccharide biosynthesis, and other beneficial traits have become accessible. Despite growing interest in postbiotics, the genomic determinants underlying the suitability of individual strains for postbiotic production are not routinely evaluated prior to inactivation, limiting the rational selection of candidate microorganisms.

In line with these considerations, the aim of the present study was to characterize *Lp. plantarum* strains DM1 and KK1 through WGS annotation and in silico assessment of their probiotic potential. To support in silico predictions, antimicrobial activity and exopolysaccharide production were validated experimentally. Lastly, multiple inactivation techniques, including ionizing *γ*-irradiation, thermal (pasteurization), enzymatic (LYZ) and mechanical treatments (US and glass-beads), as well as synergistic combinations of enzymatic treatment with ultrasound or pasteurization, were compared as strategies for the production of postbiotics.

## 2. Materials and Methods

### 2.1. Bacterial Strains and Cultivation Conditions

Two LAB strains of *Lp. plantarum* were used in this study: DM1, isolated from donkey milk and KK1, isolated from sauerkraut brine. Closely related LAB strains, *Lactobacillus* (*Lb*.) *helveticus* M92, *Lactococcus* (*Lc.*) *lactis* LMG 9450, and *Enterococcus* (*Ec.*) *faecium* ATCC^®^ 9430™ were used to evaluate the antimicrobial activity of strains DM1 and KK1. The strains were maintained as frozen stocks at −80 °C in MRS (De Man-Rogosa-Sharpe) broth (Carl Roth GmbH, Karlsruhe, Germany) for *Enterococcus* and *Lactobacillus* strains, and in M17 broth (Biolife, Milan, Italy) for the *Lactococcus* strain, with all media supplemented with 15% (*v*/*v*) glycerol (Sigma-Aldrich, Saint Louis, MO, USA). The strains were stored in the Culture Collection of the Laboratory for Antibiotic, Enzyme, Probiotic and Starter Cultures Technology, University of Zagreb, Faculty of Food Technology and Biotechnology. Prior to each experiment, strains were cultured in MRS broth and incubated at 37 °C under microaerophilic conditions overnight.

### 2.2. WGS and Functional Annotation of Lp. plantarum Genomes

Genomic DNA was isolated according to Butorac et al. [16] and sequenced on an Illumina MiSeq 2500 platform (Illumina, San Diego, CA, USA) at IGA Technology Services (Udine, Italy), using a method described by Banić et al. [17]. Sequenced genomes were deposited in NCBI database under project PRJNA1370674, with accession numbers SAMN53475367 (DM1 strain) and SAMN53475369 (KK1 strain). Functional annotation was performed using both the RAST server (Rapid Annotations using Subsystems Technology; http://rast.nmpdr.org/rast.cgi, accessed on 27 January 2026) and BV-BRC system (Bacterial and Viral Bioinformatics Resource Center, https://www.bv-brc.org/, accessed on 2 March 2026). Annotation included identification of protein-coding genes, functional assignment of predicted proteins and prediction of genomic subsystems. Circular genome maps were generated using the Circular Viewer functionality integrated into the BV-BRC system. The BAGEL4 server (http://bagel.molgenrug.nl/, accessed on 9 February 2026) was used for in silico identification of gene clusters involved in bacteriocin synthesis [18]. gutSMASH version 2.0.1. (https://gutsmash.bioinformatics.nl/, accessed on 6 March 2026) was used for specialized primary metabolite analysis in anaerobic bacteria [19], antiSMASH version 8.0.4 (https://antismash.secondarymetabolites.org/, accessed on 6 March 2026) to analyze gene clusters involved in secondary metabolite biosynthesis [20] and its pipeline epsSMASH (https://epssmash.secondarymetabolites.org/, accessed on 20 March 2026) to identify biosynthetic gene clusters that encode the biosynthetic pathways responsible for exopolysaccharide production [21]. ResFinder 4.7.2. (https://genepi.food.dtu.dk/resfinder, accessed on 11 March 2026) was used with default parameters (90% identity and 60% minimum coverage) for in silico identification of acquired genes and/or chromosomal mutations in bacterial genome mediating antimicrobial resistance [22].

### 2.3. Preparation of LAB Suspension in Dairy By-Products

Following overnight incubation in MRS medium, LAB cells were harvested by centrifugation (10 min, 4200× *g*, 4 °C) and resuspended in 50 mL of previously pasteurized (85 °C, 3 min) dairy by-product, namely sweet whey, sour whey or milk permeate. The LAB suspension was incubated in anaerobic jars under microaerophilic conditions overnight at 37 °C and subsequently subjected to inactivation treatments. All LAB suspensions were prepared and analyzed in triplicates. Chosen dairy by-products with a defined composition were obtained from DUKAT Dairy Industry Inc., Factory Bjelovar, Croatia: milk permeate (0.01% fat, 0.17% protein, and 5.65% dry matter) and whey (0.24% fat, 0.86% protein, and 6.5% dry matter).

### 2.4. Antimicrobial Activity Assay

The antimicrobial activity of strains KK1 and DM1 against closely related LAB was evaluated using the agar spot assay. Overnight cultures of KK1 and DM1 were spotted onto MRS agar plates in 10 µL aliquots and incubated under microaerophilic conditions at 37 °C for 24 h to allow colony development. Subsequently, the plates were overlaid with soft agar (0.75%, *w*/*v*) prepared in the appropriate growth medium (M17 for *Lactococcus lactis* LMG 9450, MRS for *Lactobacillus helveticus* M92 and *Enterococcus faecium* ATCC^®^ 9430™), inoculated with indicator strains adjusted to an optical density of OD_620_ = 2.0. Following incubation at 37 °C for an additional 24 h, antimicrobial activity was evaluated by measuring the colony diameter (CD) and the total diameter of the inhibition zone surrounding the colony (inhibition diameter, ID) [23]. Measurements were obtained from three independent experiments, and the effective inhibition ratio (EIR) was calculated according to the Equation (1):

(1) $$EIR=\frac{ID-CD}{CD}$$

### 2.5. Exopolysaccharide Production

Exopolysaccharide (EPS) production by strains DM1 and KK1 was evaluated phenotypically according to the method described by Cerning [24]. Briefly, bacterial strains were grown overnight on MRS agar supplemented with 2% (*w*/*v*) glucose (Kemika, Zagreb, Croatia). EPS production was assessed by gently touching bacterial colonies with a sterile inoculation loop. The formation of long, filamentous, mucoid strands characteristic of the ropy phenotype, was considered indicative of EPS production.

### 2.6. LAB Inactivation Treatments

Aliquots (1.5 mL) of previously prepared LAB suspensions were collected in triplicate and subjected to seven different cell-inactivation methods. As a control, untreated samples in the corresponding matrices were included and processed in parallel under identical conditions. Following treatment, samples were stored at 4 °C until total viable cell counts were determined using an indirect method to evaluate the LAB inactivation efficiency.

#### 2.6.1. Thermal Inactivation by Pasteurization

Samples (1.5 mL) were collected in triplicate and pasteurized at 85 °C for 3 min in a water bath.

#### 2.6.2. Enzymatic Inactivation Using Lysozyme

To obtain the LAB biomass, samples (1.5 mL) were centrifuged (4000× *g*, 10 min, 4 °C), washed with distilled water and centrifuged again (4000× *g*, 10 min, 4 °C). Enzymatic inactivation was performed by resuspending LAB biomass in 1.5 mL of lysozyme (LYZ) solution (25 mg/mL; Eurobio, Les Ulis, France) prepared in TE (Tris-EDTA) buffer (10 mM Tris–HCl, 1 mM EDTA, pH 8.0), followed by incubation at 37 °C for 15 min.

#### 2.6.3. Mechanical Inactivation Using Glass-Beads

LAB inactivation using glass-beads was achieved by adding four sterile glass-beads (d = 2 mm) to 1.5 mL of the LAB suspension held at room temperature. Samples were vertically vortexed using Biosan V-1 plus vortex, in three cycles consisting of 30 s mixing intervals followed by 15 s pauses. After treatment, glass-beads were collected for reuse and LAB suspension was further analyzed.

#### 2.6.4. Mechanical Inactivation by Ultrasound

The effect of ultrasonic treatment on LAB inactivation was evaluated using a Sonopuls Mini20 ultrasonic device (Bandelin GmbH & Co., Berlin, Germany) operated in pulse mode. LAB suspensions in whey/milk permeate (1.5 mL) were processed in open 2 mL microcentrifuge tubes with the probe positioned ~2 mm above the tube bottom. Sonication was applied in three cycles consisting of 30 s sonication intervals (frequency = 30 kHz; amplitude = 50%) followed by 15 s pauses. Samples were maintained in an ice bath throughout treatment to prevent overheating.

#### 2.6.5. Inactivation by Ionizing γ-Irradiation

Ionizing radiation experiments were conducted at the Radiation Chemistry and Dosimetry Laboratory, Ruđer Bošković Institute. Aliquots (1.5 mL) of the previously prepared bacterial suspensions were placed in 2 mL microtubes and irradiated under ambient conditions (in air, at room temperature). Preliminary radiation treatments were performed solely on the DM1 strain in a panoramic-type ^60^Co *γ*-irradiation facility at doses ranging from 0.025 to 6 kGy (0.025, 0.50, 0.75, 1.00, 2.00, 3.00, 4.00, 5.00, and 6.00 kGy) in order to determine the optimal dose for complete inactivation of bacterial cells, which was later applied on both strains. Dose mapping of the irradiation facility was previously performed experimentally using ionizing chambers and ECB dosimetric system and by simulation calculations [25].

#### 2.6.6. Combined Enzymatic and Ultrasonic Inactivation

To evaluate the synergistic effect of enzymatic and ultrasonic treatments, LAB biomass was first subjected to LYZ treatment as described in Section 2.6.2. and subsequently exposed to US under the conditions specified in Section 2.6.4.

#### 2.6.7. Combined Enzymatic and Thermal Inactivation

To evaluate the synergistic effect of enzymatic and thermal treatments, LAB biomass was first pasteurized under the conditions specified in Section 2.6.1. and subsequently subjected to LYZ treatment as described in Section 2.6.2.

### 2.7. Determination of Total Viable Bacterial Cell Counts (Indirect Method)

The total number of viable bacterial cells in control (untreated) and treated samples was determined using an indirect plate count method. Serial decimal dilutions were prepared in sterile distilled water and 10 µL aliquots of appropriate dilutions were plated in duplicate onto MRS agar plates. Inoculated MRS agar plates were incubated at 37 °C in jars under microaerophilic conditions for 48 h, after which the number of colony-forming units (CFU) was determined. All analyses were performed in triplicate.

### 2.8. Statistical Analysis

All in vitro experiments were conducted with three independent biological replicates. To assess the significance of differences between multiple pairs of means in the data group, an ordinary one-way analysis of variance (ANOVA) test was used. Statistical differences between groups were considered significant if *p*-values were less than 0.05 and then mean separation was performed with Fisher’s LSD test (http://vassarstats.net/, accessed on 20 May 2026). Significantly different means were marked with different letters. The calculated mean values and the associated statistical differences are shown in the tables and figures.

## 3. Results and Discussion

To evaluate the suitability of the newly isolated LAB strains for postbiotic production, results are organized in three consecutive steps: (i) genome-informed assessment of probiotic-associated traits in *Lp. plantarum* DM1 and KK1 strains, (ii) preliminary experimental validation of probiotic traits, and (iii) optimization of postbiotic production through LAB inactivation.

### 3.1. WGS and In Silico Evaluation of Probiotic Potential of Newly Isolated Strains

The probiotic potential of two LAB strains, isolated from donkey milk and sauerkraut brine, was assessed through genome mining and functional gene characterization to support the development of postbiotic formulations. This approach enabled the identification of probiotic-associated clusters and genes, providing a rational basis for selecting strains with desirable functional properties.

**Figure 1:**
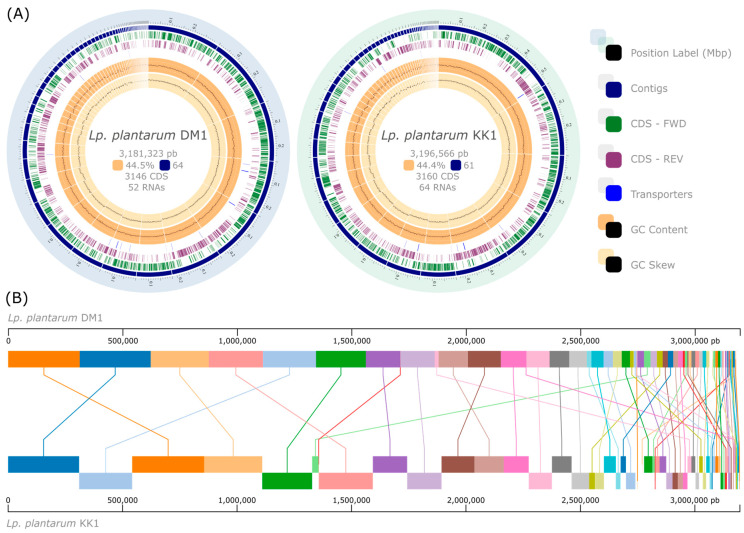
Comparative genome analysis of *Lp. plantarum* strains DM1 and KK1 obtained using the RAST server: circular genome maps (**A**) and genome alignment (**B**). Abbreviations: coding DNA sequence (*CDS*), guanine-cytosine (*GC*), forward (*FWD*) and reverse (*REV*).

First, WGS verified the taxonomic assignment of both isolated LAB strains to *Lp. plantarum*. Comparative genome analysis of strains DM1 and KK1 is summarized in Figure 1. Both strains exhibited similar genome sizes (3,181,323 and 3,196,566 bp), guanine-cytosine (GC) content (44.5 and 44.4%) and number of coding DNA sequences (CDS) and subsystems, indicating overall comparable genomic organization and conserved metabolic versatility characteristic of this species [26]. Genome alignment (Figure 1B) revealed a high level of physical colocalization of genetic loci. Most regions aligned collinearly, suggesting that conservation of gene order and the absence of major structural rearrangements indicated overall genomic stability. However, localized rearrangements and insertions were observed, particularly near terminal regions. These areas with reduced collinearity may correspond to genomic islands, mobile elements, or horizontally acquired genes, potentially reflecting adaptation to distinct ecological niches. Indeed, KK1 was isolated from sauerkraut brine, whereas DM1 originated from donkey milk. Consequently, this may account for functional differentiation between these strains, relevant to probiotic performance. Overall, these findings demonstrate that even closely related *Lp. plantarum* strains can exhibit meaningful genomic variation, underscoring the importance of detailed comparative analyses when selecting strains for biotechnological or health-related applications.

#### 3.1.1. Functional Genome Annotation and Subsystem Distribution

**Figure 2:**
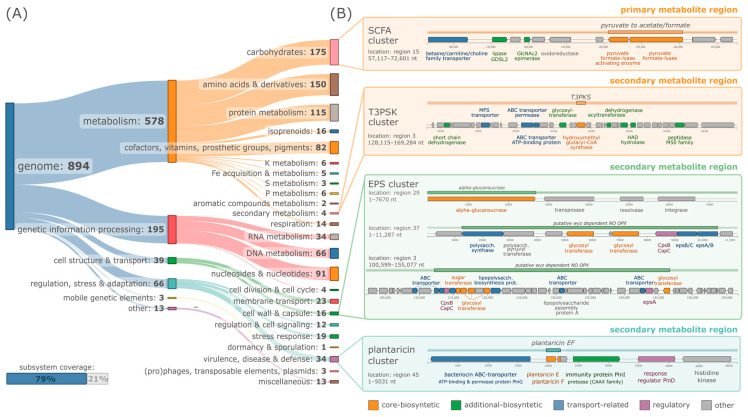
Overview of genome annotation for *Lp. plantarum* DM1 strain (**A**) with representative gene clusters highlighting its probiotic-related traits (**B**). Sankey diagram demonstrates the relative abundance of subsystem categories and the counts of each subsystem feature.

Following genome characterization, the two genomic sequences (NCBI accession numbers SAMN53475367 for DM1 and SAMN53475369 for KK1) were annotated using both the RAST server and the BV-BRC platform. Both strains exhibited highly similar distributions of functional subsystems, with a total of 894 subsystem features predicted per genome. Therefore, a detailed functional annotation is presented for the DM1 strain in Figure 2A, while the corresponding results for KK1 are provided in Figure S1. Given the high genomic similarity between the investigated *Lp. plantarum* strains and their close taxonomic relationship, the following sections focus primarily on the functional characteristics of DM1. Strain-specific genomic differences and their potential functional implications are discussed separately in a dedicated comparative analysis.

Overall, 79% of coding sequences were assigned to functional subsystems, indicating high annotation coverage. As expected, functional categorization revealed that metabolism-related genes predominated (578 genes), with carbohydrate metabolism (175 genes) together with amino acid and derivative metabolism (150 genes) representing the largest subcategories. This distribution reflects the well-known metabolic versatility of *Lp. plantarum* strains, supporting their survival in diverse environments and contributing to the production of bioactive metabolites [26,27]. Next, genes associated with genetic information processing accounted for 195 features, reflecting conserved housekeeping functions. Less represented categories such as regulation, stress response and adaptation (66 genes), including virulence, disease and defense (34 genes), are particularly relevant in the context of postbiotic applications. Notably, these genes primarily correspond to typical LAB defense mechanisms, such as bacteriocin production and stress tolerance systems, rather than pathogenic determinants. Based on this annotation, gene clusters associated with key probiotic traits were first investigated to further elucidate the functional potential of *Lp. plantarum* strains, as they represent coordinated functional units.

#### 3.1.2. Identification of Probiotic-Associated Primary and Secondary Metabolite Biosynthetic Gene Clusters

The graphical representation of identified probiotic-related gene clusters involved in primary and secondary metabolism is presented in Figure 2B, while a more detailed gene-level analysis is provided in Tables S1–S5. Notably, the strains harbor a highly conserved primary metabolic gene cluster involved in anaerobic metabolism (Figure 2B, Table S1). Located in region 15, this cluster comprises 11 genes, including those encoding for central enzyme pyruvate formate lyase, its activating enzyme and corresponding transporters. GutSMASH attributed it to the pyruvate-to-acetate/formate pathway, a key component of mixed-acid fermentation. The presence of this pathway suggests the capacity for production of short-chain fatty acids (SCFAs) such as acetate. Acetate is one of the main gut SCFA that contributes to lowering intestinal pH, thereby inhibiting pathogen growth. It also supports cross-feeding interactions within the microbiota, strengthens the epithelial barrier and modulates host immunity [28]. Therefore, the presence of this pathway is consistent with the DM1/KK1 metabolic probiotic potential. Next, five secondary metabolite regions were identified using “relaxed” strictness setting implemented in the antiSMASH platform, of which two could be linked to probiotic-related properties (Tables S2, S5 and S6). The first, a type III polyketide synthase (T3PKS) cluster located in region 3, suggests the presence of a putative secondary metabolite assembly line. However, no canonical T3PKS core enzyme was identified. Instead, the cluster contains genes encoding hydroxymethylglutaryl-CoA synthase, six tailoring enzymes (dehydrogenases, transferases, a hydrolase and a peptidase) and three transporters (ABC and MFS types), likely involved in metabolite modification and secretion. T3PKS-associated pathways are typically involved in the production of polyketides with diverse bioactive properties including antimicrobial, antioxidant and signaling functions [29]. Although the absence of a gene encoding core synthase limits functional prediction, the presence of multiple genes encoding tailoring enzymes and transport systems may represent a cryptic or incomplete T3PKS-associated locus. Since many bacterial PKS pathways remain uncharacterized [30], particularly in LAB, the identification of this cluster is noteworthy. If functional, such loci could contribute to the competitive exclusion of pathogens and enhanced ecological fitness within host-associated microbiota, which are key probiotic features. The second cluster, classified as a ribosomally synthesized and post-translationally modified peptide product (RiPP)-like region, was located in region 45 in DM1 and region 41 in KK1. As it contains genes encoding an ABC transporter, a processing enzyme (CaaX-type peptidase) and other bacteriocin-related genes, features characteristic of bacteriocin biosynthetic pathways, the region was further analyzed using BAGEL4, a tool specialized in bacteriocin identification. Notably, a plantaricin biosynthetic gene cluster was detected, including genes encoding structural peptides (PlnE and PlnF), associated immunity proteins and an ABC transporter system (LanT) involved in secretion and leader peptide processing. The presence of this cluster supports the potential of strains DM1 and KK1 for bacteriocin-mediated antagonism, a well-characterized mechanism contributing to competitive LAB fitness and microbial inhibition [31,32]. Furthermore, the identification of such defense-associated features, despite being rare and challenging to detect, is consistent with characteristics typically observed in LAB, including bacteriocin production. These bioactive compounds are particularly relevant in the context of postbiotics, as their functional properties can be retained following bacterial inactivation. Lastly, genome screening revealed three gene clusters associated with exopolysaccharide (EPS) biosynthesis in both DM1 and KK1 (Tables S3 and S4). EPSs, extracellular carbohydrate polymers with health-promoting effects [33], can be synthesized via four general pathways [34], two of which were identified in this study. The first cluster strictly follows a sucrase-dependent pathway, encoding an α-glucansucrase responsible for extracellular EPS synthesis. The same cluster also contains three accompanying genes (coding for transposase, resolvase and integrase) typically associated with mobile genetic elements. They may indirectly influence EPS biosynthesis through shaping, regulating and stabilizing the gene cluster. The remaining two clusters represent putative Wzx/Wzy-dependent pathways lacking an outer membrane porin (OPX). As the most widespread EPS biosynthesis pathway in bacteria, it is facilitated by a series of glycosyltransferases that generate EPSs with diverse composition [34,35,36]. The high variability of such clusters, even among closely related strains, may explain the relatively low sequence similarity observed (up to 30% by BLAST analysis).

It is worth mentioning that KK1 genome mining identified the same probiotic-associated primary and secondary metabolite biosynthetic gene clusters (data shown in Supplementary Materials).

#### 3.1.3. Genes Associated with Probiotic Functionality

Altogether, the genetic clusters annotated to metabolic and biosynthetic pathways support the multifunctional probiotic potential of DM1 and KK1, particularly in terms of metabolite production, antimicrobial activity and host–bacterial interactions. Based on these findings, further genome mining identified additional key genes located outside biosynthetic clusters associated with probiotic-related functional traits. The results are summarized in Table 1, highlighting genes encoding proteins associated with gastrointestinal stress tolerance, adhesion to intestinal epithelial surfaces, prebiotic substrate utilization and antimicrobial production.

Table 1 demonstrates a diverse repertoire of genes associated with probiotic functionalities in both strains, as identified through BV-BRC feature screening. First, genes related to gastrointestinal stress tolerance were well represented, including those associated with acid and bile salts resistance (bile salt hydrolases and glutamate decarboxylase), oxidative stress response (glutaredoxin, thioredoxin reductase, catalase, ClpP protease) and heat shock response (GroES, GroEL, HtpX, GrpE, DnaK/DnaJ chaperone systems). These mechanisms are responsible for maintaining cellular homeostasis and contributing to bacterial survival under harsh gastrointestinal conditions (low pH and oxidative stress) encountered during passage through the human digestive tract [37]. The presence of multiple complementary stress-response mechanisms suggests that both strains possess a degree of resilience to gastrointestinal challenges. Another key probiotic trait is the bacterial ability to adhere to the intestinal epithelium, which supports persistence, colonization and host interaction. Although genes encoding mucus-binding proteins were not detected, several genes associated with adhesion and surface interactions were identified. These include genes encoding fibronectin-binding proteins, teichoic and lipoteichoic acid components, as well as previously defined EPSs, all of which may contribute to surface attachment and host–microbe interactions. Interestingly, the absence of a gene encoding a teichoic acid glycosylation protein in KK1, present in DM1, may suggest potential differences in cell wall decoration patterns. Next, the ability to utilize diverse prebiotic substrates and modulate gut environment represents another important probiotic-related trait [38]. Having inherent genes for metabolism of oligosaccharides (α-galactosidase), pyruvate (pyruvate oxidase, pyruvate formate-lyase and pyruvate dehydrogenase complex) and lactate (D- and L-lactate dehydrogenase), LAB can ferment alternative energy sources. This may support microbiota modulation through metabolic cross-feeding and substrate utilization [39]. Finally, based on the presence of genes associated with plantaricin EF and acetate production, DM1 and KK1 antimicrobial production potential was inferred.

Lastly, the safety profile of LAB strains intended for postbiotic applications must be evaluated before potential use. A strain is generally considered safe if it lacks virulence factors and transferable antimicrobial resistance (AMR) genes, such as *tet* (tetracycline), *erm* (macrolide), *van* (vancomycin) or *bla* (*β*-lactam). Genome analysis performed using the ResFinder platform revealed no acquired AMR genes or antimicrobial resistance-associated chromosomal mutations in either strain. These findings were further supported by BV-BRC-based analysis which did not identify known virulence determinants and predicted that both bacterial genomes are non-pathogenic to humans. These analyses additionally supported the suitability of DM1 and KK1 for further evaluation in postbiotic applications.

Altogether, the genome-based analyses indicate that both strains possess a broad range of genes connected with probiotic-associated traits, including metabolic versatility, antimicrobial potential, stress tolerance and host interaction. Importantly, many of these functional traits are associated with cellular components and metabolites (such as SCFAs, bacteriocins and EPSs) that can retain biological activity even after cell inactivation and can function independently as postbiotics. As these findings are based on in silico predictions, they provide a strong basis for experimental validation of functional activity, which we plan to address in subsequent research. Accordingly, the next phase of this study focuses on evaluating different inactivation strategies to generate stable, non-viable postbiotic formulations while preserving their functional properties.

### 3.2. Preliminary Experimental Validation of Probiotic Traits

The genomic analyses presented above provide valuable insights into the functional potential of the investigated strains; however, they do not confirm the actual expression of predicted traits. As in silico analyses alone are insufficient to establish probiotic functionality, preliminary experimental assays were performed to evaluate antimicrobial activity and EPS production in DM1 and KK1. First, antimicrobial activity of DM1 and KK1 strains against closely related LAB was evaluated using an agar spot method and expressed as effective inhibition ratio (EIR) as shown in Table 2. According to Coeuret et al. [23], EIR values < 0.5 indicate weak inhibition, values between 0.5 and 1.5 indicate moderate inhibitory activity, while EIR values > 1.5 indicate strong inhibition. As seen from the data obtained, both strains exhibited moderate to strong inhibitory activity against the tested indicator strains. While KK1 displayed relatively consistent inhibitory effects across all tested LAB, DM1 showed significantly greater variability in inhibition strength depending on the target strain.

Secondly, since EPSs can contribute to bacterial surface interactions and adhesion-related properties, DM1 and KK1 were evaluated for EPS production. Previously identified EPS biosynthesis glycosyltransferase EpsF through genome mining was confirmed by detecting a ropy phenotype in both strains (Figure S2). This physical characteristic is visible as long and stringy secretions, indicative of dense EPS production. However, further analyses are needed to validate the composition of these valuable probiotic metabolites. These preliminary findings provide experimental support for selected probiotic-associated traits predicted by genome mining and justify the inclusion of DM1 and KK1 in subsequent postbiotic production experiments. Nevertheless, comprehensive functional characterization, including metabolomic, proteomic, and additional physiological analyses, will be required to further validate their probiotic potential and the functional properties of the resulting postbiotics.

### 3.3. Evaluation of LAB Inactivation Strategies for Postbiotic Preparation

Unlike probiotics, postbiotics consist of non-viable microbial cells, their structural components and metabolites that can confer health benefits to the host [5]. Controlled bacterial inactivation is therefore a critical step in postbiotic production, as the applied treatment must ensure complete loss of cell viability while preserving beneficial microbial components. To identify suitable approaches for postbiotic generation, several LAB inactivation methods were investigated, including thermal (pasteurization), enzymatic (LYZ), mechanical (glass-beads and US) and radiation-based treatments (ionizing *γ*-irradiation). In addition, combined approaches involving enzymatic treatment followed by thermal or ultrasonic inactivation were also evaluated.

#### 3.3.1. Optimization of γ-Irradiation for *Lp. plantarum* Inactivation

Gamma irradiation was first examined due to its well-established ability to achieve complete microbial inactivation while avoiding thermal degradation of cellular components. Hojjati et al. showed that γ-irradiation doses of 5–15 kGy improve the antioxidant activity and prebiotic potential of polysaccharides by modifying physicochemical characterization without changing functional groups [40]. Furthermore, Correa et al. showed that γ-irradiation doses >5.5 kGy damage DNA and generate reactive oxygen species (ROS) through interaction with water, which in turn causes ROS-induced protein oxidation [41]. Therefore, bactericidal effects of γ-irradiation depend on the level of antioxidants and the DNA repair activity of the bacterial cells. This method is widely used in food systems for microbial decontamination, effectively eliminating foodborne bacteria while causing minimal changes in physicochemical or sensory properties [42]. Furthermore, *γ*-radiation doses <10 kGy are considered safe and do not induce toxicity or significant nutrient loss [43]. However, its application for postbiotic production remains limited, with reports mainly focusing on a limited number of bacterial species, including *Lactobacillus* (*Lb. acidophilus*, *Lb. casei* and *Lb. reuteri*) [44,45] and *Bifidobacterium* (*B. animalis* and *B. lactis*) [44].

**Figure 3:**
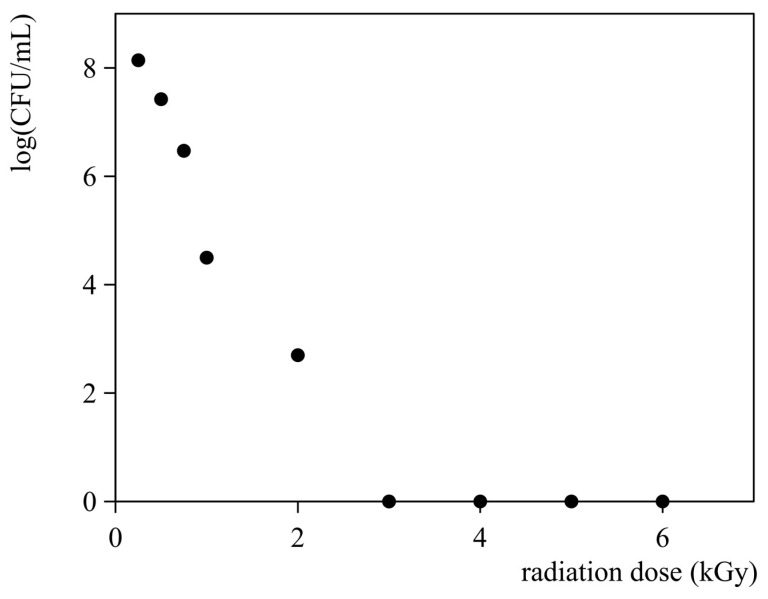
Effect of ionizing *γ*-radiation dose on viable cell count (log CFU/mL) of the DM1 strain. Data are presented as mean ± SD of three independent experiments. Error bars are included but may not be visible due to the low variability among replicates.

**Figure 4:**
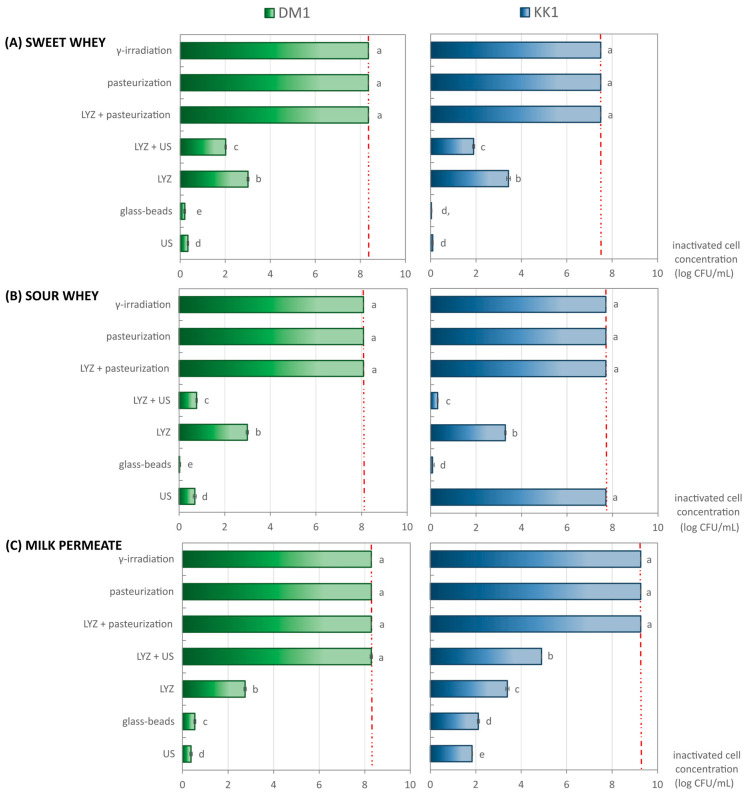
Effect of different inactivation treatments on reduction in viable cell counts (log CFU/mL) of *Lp. plantarum* strains DM1 and KK1 suspended in dairy by-product matrices: sweet whey (**A**), sour whey (**B**) and milk permeate (**C**). Bars represent the inactivated bacterial concentrations following treatment. The red dashed line indicates the initial viable cell concentration prior to treatment (log CFU/mL). Abbrev.: lysozyme (LYZ), ultrasound (US). Different letters indicate statistically significant reduction (*p* < 0.05) of viable cell counts (log CFU/mL) compared to the non-treated control.

To determine the optimal dose for *Lp. plantarum* inactivation, nine *γ*-irradiation doses were applied to the DM1 strain and *γ*-radiation survival curve was obtained (Figure 3). The DM1 strain was selected as a representative strain for this experiment, as similar radiation resistance was expected among the investigated *Lp. plantarum* strains due to their close taxonomic relationship, high genomic similarity and comparable functional profiles identified in Section 3.1. Increasing *γ*-irradiation doses (up to 3 kGy) caused a linear reduction in viable cell counts (log CFU/mL). More precisely, lower doses caused only partial bacterial inactivation and were insufficient to eliminate all viable cells. At the lowest tested dose (0.25 kGy), cell counts decreased by approximately one log unit compared to the untreated control. With increasing radiation dose, the inactivation effect became progressively more pronounced, reaching a reduction of approximately seven log units at 2 kGy. Complete bacterial inactivation was achieved at 3 kGy, with no viable cells detected at higher doses (4–6 kGy). These findings demonstrate that relatively low irradiation doses are sufficient for complete DM1 inactivation, supporting its feasibility as a non-thermal strategy for postbiotic production. The observed dose-dependent reduction in viability is consistent with the known antimicrobial mechanism of ionizing radiation, including DNA damage, oxidative stress, and disruption of cellular structures. Based on these results, 3 kGy was selected as the threshold dose for subsequent postbiotic production experiments, including applications in dairy by-product matrices. The applicability of this dose was further confirmed for strain KK1, which exhibited complete inactivation under the same conditions (Figure 4).

#### 3.3.2. Matrix-Dependent Evaluation of LAB Inactivation Treatments

Although *γ*-irradiation enabled efficient, rapid and complete inactivation of *Lp. plantarum*, the suitability of alternative approaches was also examined to identify more accessible, practical and cost-effective methods suitable for routine laboratory application and potential large-scale postbiotic production. Since postbiotic formulations are often produced in complex food matrices, it is important to assess the effectiveness of inactivation strategies under such conditions. Food components such as proteins, carbohydrates and minerals may exert protective effects on bacterial cells and potentially reduce inactivation efficiency [46]. Moreover, it has been suggested that lactose interacts with the cell membrane and helps to maintain bacterial membrane integrity, while whey proteins, due to their gelation properties, may also have a protective effect on bacterial cells. However, a high level of milk fat may have a detrimental effect, and protective effects during convective droplet drying of LAB cells have been attributed to calcium and milk proteins rather than lactose [47,48]. Sweet whey, sour whey and milk permeate were selected as model matrices because they represent abundant dairy by-products with potential applications as sustainable and cost-effective substrates for postbiotic production. In addition, their differing compositions provide an opportunity to evaluate matrix-dependent effects on bacterial inactivation. Therefore, the selected *Lp. plantarum* strains DM1 and KK1 were resuspended in these three dairy by-products and subjected to thermal, enzymatic, mechanical, radiation-based (3 kGy) and combined inactivation treatments. The comparative effects of these treatments on viable cell counts are presented in Figure 4.

As shown in Figure 4, treatment efficacy varied substantially depending on both the applied inactivation method and the dairy matrix. Thermal treatment (pasteurization) and ionizing *γ*-irradiation (3 kGy) efficiently inactivated both *Lp. plantarum* strains (DM1 and KK1) across all tested dairy matrices. As expected, a similar outcome to pasteurization alone was observed for the combined LYZ-pasteurization treatment, which also eliminated statistically significant levels of viable cells. In contrast, all other treatments achieved only partial reductions in bacterial viability.

Milder mechanical treatments (glass-beads and US) were tested for their ability to disrupt cell wall integrity and reduce viable cell counts. Glass-bead disruption had minimal impact, with reductions generally below one log unit, indicating that this mechanical treatment was insufficient for effective LAB inactivation. In contrast, the US exhibited strong matrix-dependent variability. While complete inactivation of KK1 strain was observed only in sour whey, substantially lower reductions were detected for both strains in sweet whey and milk permeate, where viable cell counts remained close to control levels in several cases, similar to glass-bead treatment. As the US acts via cavitation bubbles that disrupt the cell membrane, its effectiveness depends on treatment intensity and duration [49]. Therefore, the observed variability likely reflects differences in matrix composition that can influence cavitation efficiency and bacterial susceptibility to mechanical disruption.

Lastly, enzymatic treatment with LYZ, which hydrolyses the peptidoglycan layer of the LAB cell wall, was previously reported to be highly effective in the disruption of LAB strains [50]. In all LYZ-treated samples, moderate reductions of approximately three–four log units were observed, consistent across matrices. While this confirms LYZ antimicrobial activity, the enzymatic treatment alone is insufficient for complete inactivation. The combination of LYZ and US showed an enhanced effect compared with either treatment alone only in milk permeate, where viable counts decreased approximately five log units. This synergistic effect combines enzymatic degradation of the cell wall with mechanical disruption of the membrane [10]. However, this effect was not observed in whey-based matrices, indicating a potential protective role of matrix components. In contrast, the combination of LYZ and pasteurization exhibited the strongest inactivation effect, indicating that thermal treatment plays a critical role in achieving complete microbial inactivation. This enhanced lethality reflects the synergistic interaction between enzymatic cell wall degradation and thermal stress, whereby LYZ-mediated weakening of the peptidoglycan layer increases bacterial susceptibility to heat-induced membrane damage (fluidization, loss of proton motive force, protein denaturation, and irreversible disruption of cellular homeostasis). Similar synergistic effects between cell wall-targeting enzymes and mild thermal treatments have been reported, where prior envelope destabilization enhances thermal sensitivity and accelerates inactivation [51,52].

#### 3.3.3. Implications for Postbiotic Formulation Development

The inactivation results indicate that only ionizing radiation (3 kGy), pasteurization and the combined LYZ-pasteurization treatment achieved consistent and considerable inactivation of both LAB strains across all tested matrices. These methods therefore appear most suitable for the production of DM1 and KK1 postbiotic formulations, balancing efficacy and practical applicability. However, the retention and biological activity of postbiotic-associated bioactive compounds following inactivation were not evaluated in the present study and remain important considerations for postbiotic development. Future studies should therefore investigate the preservation, stability and functionality of key bioactive components after exposure to different inactivation treatments. Moreover, the obtained results highlight the importance of matrix-dependent effects when selecting inactivation approaches and provide valuable insights for the development of postbiotic functional products containing non-viable LAB and their bioactive components.

## 4. Conclusions

This study presents a genome-guided approach for the identification and in silico characterization of *Lp. plantarum* strains DM1 and KK1 with potential applications in postbiotic development. WGS confirmed their taxonomic identity and revealed a conserved genomic framework with localized variations that may contribute to strain-specific functional traits. Genome mining identified key genetic determinants associated with probiotic traits, including metabolic versatility, antimicrobial potential (particularly plantaricin biosynthesis), stress tolerance (acid, oxidative and heat-shock stress) and putative adhesion-related functions. Overall, these findings support the probiotic potential of both strains, although further in vitro and in vivo extensive validation is required. Preliminary in vitro assays corroborated the in silico predictions, demonstrating antimicrobial activity and exopolysaccharide production in both strains.

In parallel, this study also demonstrated that the efficiency of LAB inactivation strategies is strongly influenced by both the applied treatment and the surrounding matrix. Among the evaluated approaches, thermal treatment (3 min pasteurization), ionizing *γ*-irradiation (3 kGy), and combined LYZ-pasteurization treatment consistently achieved complete inactivation of *Lp. plantarum* across all tested dairy by-products, indicating their suitability for postbiotic production. In contrast, mechanical (US and glass-beads) and enzymatic (LYZ) treatments alone were less effective and exhibited pronounced matrix-dependent variability, highlighting the protective role of food components.

Overall, these findings highlight the importance of integrating genome-based strain characterization with robust inactivation strategies effective in complex food systems. This study provides a foundation for developing stable, non-viable postbiotic-based functional products, while emphasizing the need for further studies to evaluate the preservation of bioactive compounds and their functional effects.

## Figures and Tables

**Table 1:** Overview of DM1 and KK1 genome mining for genes associated with functional probiotic properties.

Probiotic-Related Trait		Representative Protein	Gene Locus: Start–End (Strand)	
			DM1	KK1
Gastrointestinal stress tolerance	Acid and bile salts tolerance	Bile salt hydrolase (EC 3.5.1.24)	3 copies	3 copies
		Glutamate decarboxylase, Gad (EC 4.1.1.15)	4535–5944 (+)	25,851–27,260 (+)
	Oxidative stress	Glutaredoxin-like protein NrdH	69,706–69,936 (−)	166,306–166,536 (+)
		Thioredoxin reductase TrxR (EC 1.8.1.9)	2 copies	2 copies
		Tyrosine-protein phosphatase (EC 3.1.3.48)	61,930–62,721 (+)	61,930–62,721 (+)
		Clp protease, proteolytic subunit ClpP (EC 3.4.21.92)	149,442–150,032 (−)	86,210–86,800 (+)
		Glutathione biosynthesis bifunctional protein GshF (EC 6.3.2.2)	66,299–67,537 (+)	66,299–67,537 (+)
		Catalase CatE (EC 1.11.1.6)	64–1386 (−)	64–1386 (−)
	Heat shock response	Heat shock protein 10 kDa family chaperone GroES	93,680–93,964 (+)	142,278–142,562 (−)
		Heat shock protein 60 kDa family chaperone GroEL	94,020–95,645 (+)	140,597–142,222 (−)
		Heat shock protein HtpX	4563–5462 (−)	19,932–20,831 (+)
		Heat shock protein GrpE	184,282–184,890 (+)	185,890–186,498 (+)
		Chaperone protein DnaK	184,934–186,802 (+)	186,542–188,410 (+)
		Chaperone protein DnaJ	186,904–188,046 (+)	188,512–189,654 (+)
Adhesion potential	Cell surface structure modification	Teichoic acid glycosylation protein	70,435–70,956 (+)	x
		Teichoic acid export ATP-binding protein TagH (EC 3.6.3.40)	33,574–34,665 (−)	16,671–17,762 (−)
		Teichoic acid translocation permease protein TagG	34,678–35,493 (−)	17,775–18,590 (−)
		Lipoteichoic acid synthase LtaS Type IIa	25,670–27,775 (+)	243,508–245,613 (+)
		Lipoteichoic acid synthase LtaS Type IIb	181,360–183,514 (−)	181,409–183,562 (−)
	Extracellular matrix binding	Fibronectin/fibrinogen-binding protein	2 copies	2 copies
	Surface anchoring	Sortase A, LPXTG specific—srtA	6143–6847 (−)	18,547–19,251 (+)
	Biofilm formation	Exopolysaccharide biosynthesis glycosyltransferase EpsF (EC 2.4.1.)	7863–8603 (+)	3512–4252 (−)
		Aggregation promoting factor	5 copies	5 copies
Prebiotic utilization	Lactate metabolism	(D)-lactate dehydrogenase (EC 1.1.1.28)	152,272–153,270 (+)	2 copies
		(L)-lactate dehydrogenase (EC 1.1.1.27)	4 copies	6 copies
	Pyruvate metabolism	Pyruvate oxidase (EC 1.2.3.3)	5 copies	4 copies
		Pyruvate formate-lyase (EC 2.3.1.54)	67,975–70,233 (−)	47,097–49,355 (−)
		Pyruvate dehydrogenase complex *	*	*
	Carbohydrate utilization	*α*-galactosidase (EC 3.2.1.22)	115,606–117,621 (−)	2 copies
Antimicrobial production	Bacteriocin production	Plantaricin EF	1970–2314 (+)	2820–3165 (−)
	Acetate production	Acetate kinase (EC 2.7.2.1)	3 copies	3 copies

* Pyruvate dehydrogenase complex composed of the following components: dihydrolipoamide dehydrogenase (EC 1.8.1.4) and *α*- and *β*-subunit of the E1 component (EC 1.2.4.1) in both strains, with an additional dihydrolipoamide acetyltransferase component (EC 2.3.1.12) identified only in DM1 strain.3.1.4. Safety assessment and potential relevance for postbiotic applications. x = no gene detected.

**Table 2:** Antimicrobial activity of DM1 and KK1 strains against closely related LAB.

Strain	Effective Inhibition Ratio		
	*Lb. helveticus* M92	*Lc. lactis* LMG 9450	*Ec. faecium* ATCC^®^9430^TM^
DM1	3.45 ± 0.60 ^a^	0.87 ± 0.01 ^c^	1.95 ± 0.16 ^b^
KK1	0.95 ± 0.19	1.22 ± 0.10	2.11 ± 1.02

Different superscript letters indicate statistically significant differences among data within the same row (*p* < 0.05).

## Data Availability

Sequenced genomes are deposited in NCBI database under project PRJNA1370674, with accession numbers SAMN53475367 (DM1 strain) and SAMN53475369 (KK1 strain). The original contributions presented in this study are included in the article/Supplementary Materials. Further inquiries can be directed to the corresponding author.

## Supplementary Materials

The following supporting information can be downloaded at https://www.mdpi.com/article/10.3390/foods15122148/s1. Figure S1: Overview of genome annotation for *Lp. plantarum* KK1 strain; Figure S2: Ropy phenotype of *Lp. plantarum* strains (A) DM1 and (B) KK1; Table S1: ClusterBLAST scores (gutSMASH) for NODE_15 of *Lp. plantarum* strains (A) DM1 and (B) KK1; Table S2: ClusterBLAST scores (antiSMASH) for NODE_3 of *Lp. plantarum* strains (A) DM1 and (B) KK1; Table S3: ClusterBLAST scores (epsSMASH) for *Lp. plantarum* strain DM1 (A) NODE 3, (B) NODE 29 and (C) NODE 37; Table S4: ClusterBLAST scores (epsSMASH) *for Lp. plantarum* strain KK1 (A) NODE 3, (B) NODE 34 and (C) NODE 42; Table S5: ClusterBLAST scores for *Lp. plantarum* strain DM1 at NODE_45 using (A) antiSMASH and (B) BAGEL4; Table S6: ClusterBLAST scores for *Lp. plantarum* strain KK1 at NODE_41 using (A) antiSMASH and (B) BAGEL4.
