# Supplementary material for: Genomic Insights and Inactivation Strategies for Lactiplantibacillus plantarum Postbiotics Production
Source: Foods. 2026 Jun 14;15(12):2148. doi: 10.3390/foods15122148 (PMC13298353; doi:10.3390/foods15122148)

Article

Supplementary materials

Genomic Insights and Inactivation Strategies for *Lactiplanti-bacillus plantarum* Postbiotics Production

Mia Radović, Tomislava Grgić, Martina Banić, Katarina Butorac, Andreja Leboš Pavunc, Jagoda Šušković, Jasna Novak * and Blaženka Kos

Department of Biochemical Engineering, University of Zagreb Faculty of Food Technology and Biotechnology, Pierottijeva 6, 10000 Zagreb, Croatia

* Correspondence: jasna.novak@pbf.unizg.hr

This file contains all the supplementary materials for the article Genomic Insights and Inactivation Strategies for *Lactiplantibacillus plantarum* Postbiotics Production. Short summary of the materials obtained:

- **Figure S1.** Overview of genome annotation for *Lp. plantarum* KK1 strain.
- **Figure S2.** Ropy phenotype of *Lp. plantarum* strains DM1 (A) and KK1 (B).
- **Table S1.** ClusterBLAST scores (gutSMASH) for NODE_15 of *Lp. plantarum* strains (A) DM1 and (B) KK1.
- **Table S2.** ClusterBLAST scores (antiSMASH) for NODE_3 of *Lp. plantarum* strains (A) DM1 and (B) KK1.
- **Table S3.** ClusterBLAST scores (epsSMASH) for *Lp. plantarum* strain DM1 (A) NODE 3, (B) NODE 29 and (C) NODE 37.
- **Table S4.** ClusterBLAST scores (epsSMASH) *for Lp. plantarum* strain KK1 (A) NODE 3, (B) NODE 34 and (C) NODE 42.
- **Table S5.** ClusterBLAST scores for *Lp. plantarum* strain DM1 at NODE_45 using (A) antiSMASH and (B) BAGEL4.
- **Table S6.** ClusterBLAST scores for *Lp. plantarum* strain KK1 at NODE_41 using (A) antiSMASH and (B) BAGEL4.

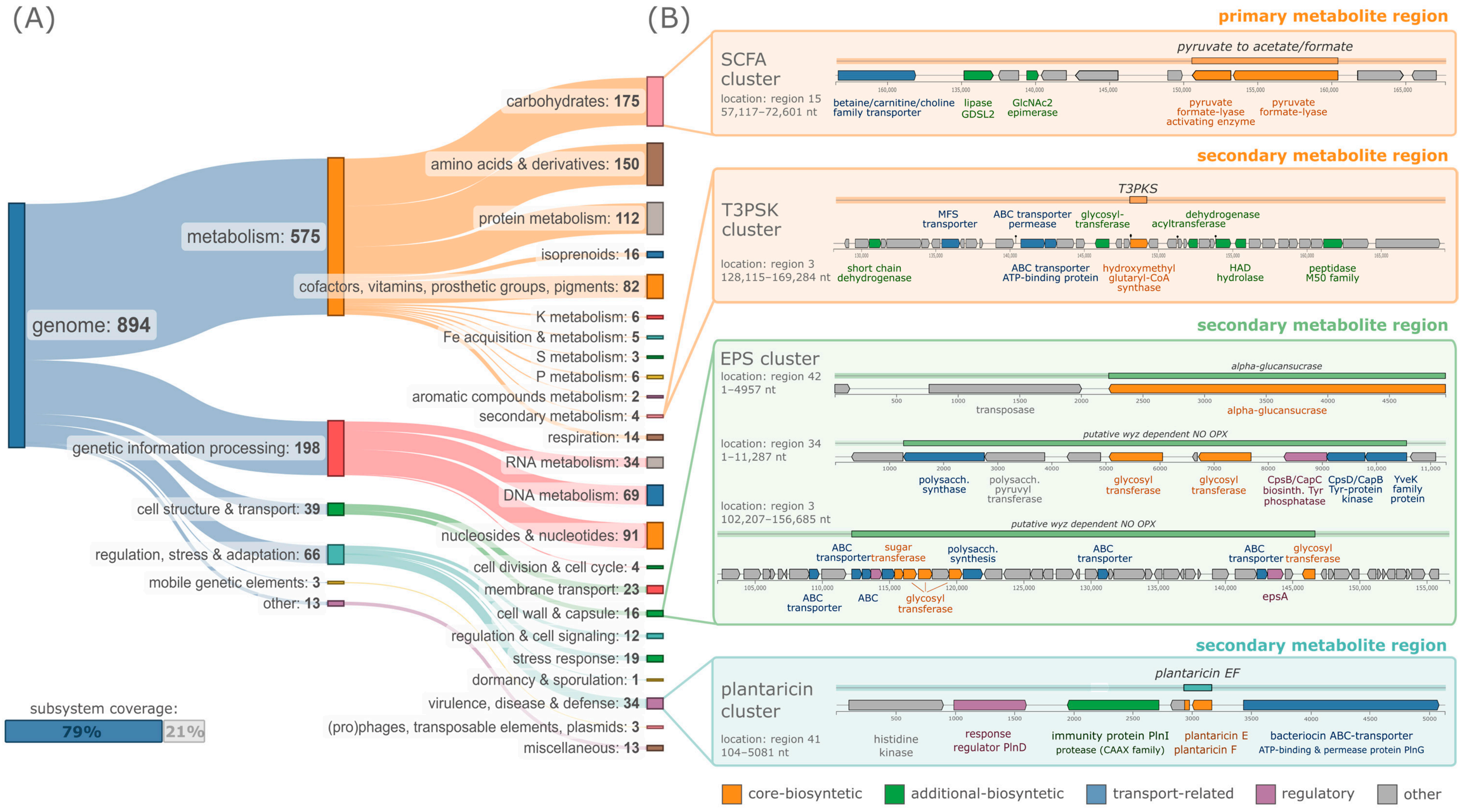

Figure S1. Overview of genome annotation for *Lp. plantarum* KK1 strain (A) with representative gene clusters highlighting its probiotic-related traits (B). Sankey diagram demonstrates the relative abundance of subsystem categories and the counts of each subsystem feature.

Figure S2. Ropy phenotype of *Lp. plantarum strains* DM1 (A) and KK1 (B).

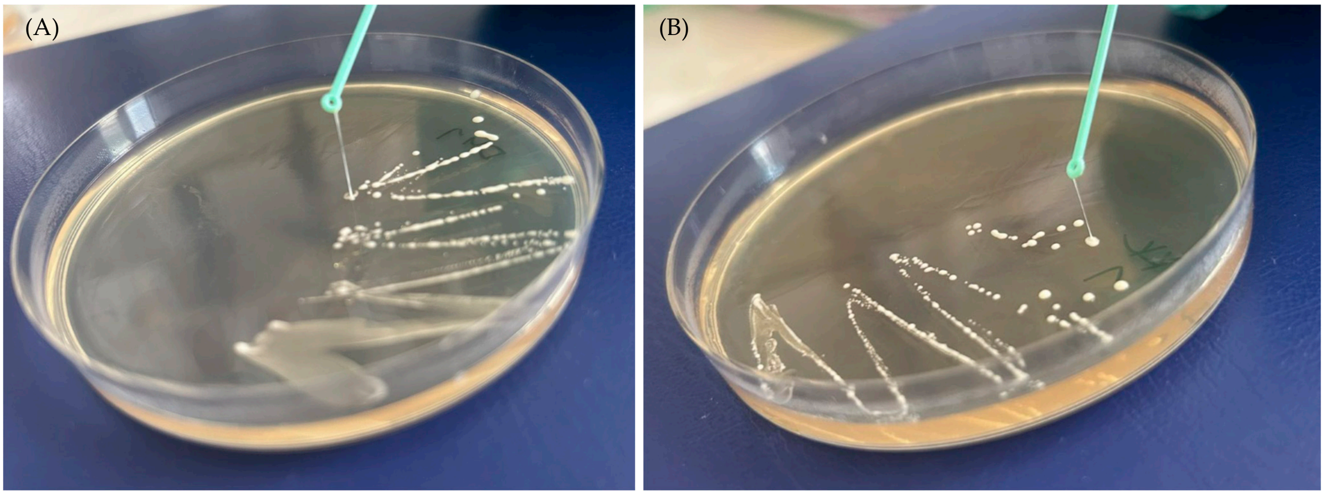

Table S1. Identified primary metabolic regions in *Lp. plantarum* strains with gutSMASH and their corresponding ClusterBLAST scores.

(A) DM1 strain with NODE_15 hit

**significant hit**	GL379766.1 *Lactobacillus plantarum* subsp. *plantarum* ATCC 14917 genomic scaffold SCAFFOLD6, whole genome shotgun sequence						
**cluster class**	**SCFA (Short Chain Fatty Acids)**						
**cluster type**	**Pyruvate2acetate-formate**						
**gene (locus tag)**	**locus**			**annotation**			
	**start (nt)**	**finish (nt)**	**strand**	**% identity**	**blast score**	**% coverage**	**e-value**
ctg15_62	59540	61208	+	100	1058	100.0	$4.8e^{-308}$
ctg15_63	62223	62865	+	98	436	100.0	$3.5e^{-121}$
ctg15_64	62967	63396	–	100	295	100.0	$6.3e^{-79}$
ctg15_65	63568	63814	+	100	162	100.0	$3.7e^{-39}$
ctg15_66	63908	64433	–	99	356	100.0	$2.2e^{-97}$
ctg15_67	64602	65523	–	99	613	100.0	$2.3e^{-174}$
ctg15_68	66582	66894	+	99	211	100.0	$8.7e^{-54}$
ctg15_69	67116	67938	–	100	572	100.0	$3e^{-162}$
ctg15_70	67974	70233	–	100	1531	100.0	0.0
ctg15_71	70650	71640	+	100	686	100.0	$2.3e^{-196}$
ctg15_72	71807	72323	–	98	350	100.0	$2e^{-95}$
Number of proteins with BLAST hits to this cluster: **11**							
Cumulative BLAST score: **6270**							

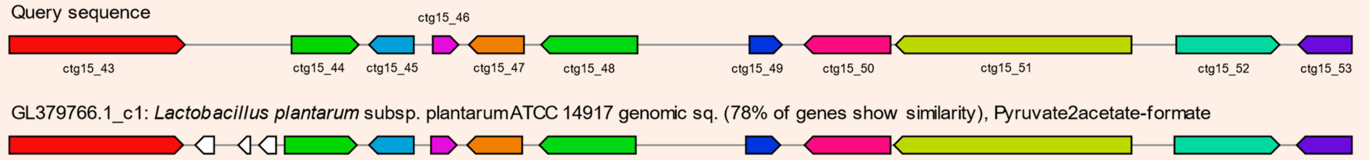

(B) KK1 strain with NODE_15 hit

**significant hit**	QRHF01000012.1 *Lactobacillus plantarum* strain AM25-20AC AM25-20AC.Scaf12, whole genome shotgun sequence						
**cluster class**	**SCFA (Short Chain Fatty Acids)**						
**cluster type**	**Pyruvate2acetate-formate**						
**gene (locus tag)**	**locus**			**annotation**			
	**start (nt)**	**finish (nt)**	**strand**	**% identity**	**blast score**	**% coverage**	**e-value**
ctg15_43	38662	40330	+	100	1058	100.0	$4.8e^{-308}$
ctg15_45	41345	41987	+	100	441	100.0	$1.1e^{-122}$
ctg15_46	42089	42518	–	99	293	100.0	$2.4e^{-78}$
ctg15_47	42690	42936	+	100	162	100.0	$3.7e^{-39}$
ctg15_48	43030	43555	–	100	360	100.0	$1.5e^{-98}$
ctg15_49	43724	44645	–	99	613	100.0	$1.7e^{-174}$
ctg15_50	45686	46016	+	99	210	94.495	$1.2e^{-53}$
ctg15_51	45998	46223	–	99	149	100.0	$2.2e^{-35}$
ctg15_52	46238	47060	–	100	572	100.0	$3e^{-162}$
ctg15_53	47096	49355	–	100	1531	100.0	0.0
ctg15_54	49772	50762	+	100	686	100.0	$2.3e^{-196}$
ctg15_55	50929	51445	–	99	353	100.0	$2.4e^{-96}$
Number of proteins with BLAST hits to this cluster: **12**							
Cumulative BLAST score: **6428**							

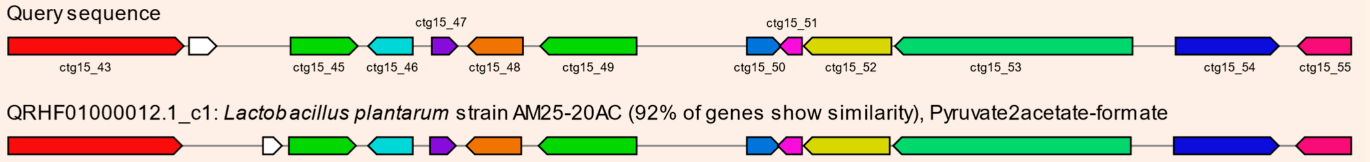

Table S2. Identified secondary metabolic regions in *Lp. plantarum* strains with antiSMASH and their corresponding ClusterBLAST scores. Significant hit for NODE_3 linked to NZ_VBSY01000002 *Lactiplantibacillus plantarum* strain FAM 21789 FAM21789_scf0002, whole genome shotgun sequence.

(A) DM1 strain: NODE_3

**cluster type**	**T3PKS** (Type III PolyKetide Synthase)						
**gene (locus tag)**	**locus**			**annotation**			
	**start (nt)**	**finish (nt)**	**strand**	**% identity**	**blast score**	**% coverage**	**e-value**
ctg3_119	127260	127545	–	100	188	100.0	$5.93e^{-62}$
ctg3_120	127977	128913	+	100	629	100.0	$2.23e^{-229}$
ctg3_121	128922	129714	+	100	501	100.0	$2.21e^{-180}$
ctg3_122	129726	130077	+	100	203	100.0	$3.32e^{-67}$
ctg3_123	130098	132435	+	100	1492	100.0	0.0
ctg3_124	132453	132972	+	100	343	100.0	$4.74e^{-121}$
ctg3_125	133114	133690	–	100	369	100.0	$2.47e^{-130}$
ctg3_126	133805	135017	+	100	735	100.0	$2.45e^{-268}$
ctg3_127	135034	135322	–	99	160	86.32	$1.14e^{-50}$
ctg3_128	135433	136195	+	98	504	100.0	$8.76e^{-182}$
ctg3_129	136370	136577	+	100	140	100.0	$7.78e^{-44}$
ctg3_130	137442	138654	+	99	798	100.0	$3.53e^{-293}$
ctg3_131	139132	140737	+	86	776	90.82	$8.6e^{-281}$
ctg3_132	140755	141547	+	100	510	100.0	$4.86e^{-184}$
ctg3_133	141551	142712	+	100	766	100.0	$5.95e^{-281}$
ctg3_134	142863	143436	+	99	367	100.0	$6.55e^{-130}$
ctg3_135	144135	145077	–	100	620	100.0	$5.89e^{-226}$
ctg3_136	145522	145915	–	97	249	100.0	$6.62e^{-85}$
ctg3_137	146078	146471	+	99	283	100.0	$1.23e^{-98}$
ctg3_138	146506	147676	+	97	743	100.0	$7.4e^{-272}$
ctg3_139	147725	148355	–	97	340	100.0	$1.29e^{-118}$
ctg3_140	148978	149611	–	100	416	100.0	$1.85e^{-148}$
ctg3_141	149729	150002	+	99	143	87.78	$1.6e^{-44}$
ctg3_142	150105	150336	+	99	143	100.0	$8.7e^{-45}$
ctg3_143	150392	151028	–	99	422	100.0	$5.12e^{-151}$
ctg3_144	151139	151898	+	100	492	100.0	$3.04e^{-177}$
ctg3_145	151881	152187	+	100	197	100.0	$2.15e^{-65}$
ctg3_146	152271	153270	+	100	654	100.0	$2.01e^{-238}$
ctg3_147	153559	154282	–	100	480	100.0	$9.11e^{-173}$
ctg3_148	154506	155310	+	100	514	100.0	$1.98e^{-185}$
ctg3_149	155412	156291	+	100	538	100.0	$6.85e^{-194}$
ctg3_150	156490	157213	+	100	459	100.0	$1.1e^{-164}$
ctg3_151	157214	157778	+	100	319	100.0	$5.93e^{-111}$
ctg3_152	157897	158677	+	100	527	100.0	$6.97e^{-191}$
ctg3_153	158692	159478	+	100	493	100.0	$3.01e^{-177}$
ctg3_154	159515	160793	+	100	802	100.0	$5.9e^{-294}$
ctg3_155	160832	162542	+	100	1117	100.0	0.0
ctg3_156	163035	167349	+	100	2860	100.0	0.0
Number of proteins with BLAST hits to this cluster: **38**							
Cumulative BLAST score: **21546.0**							

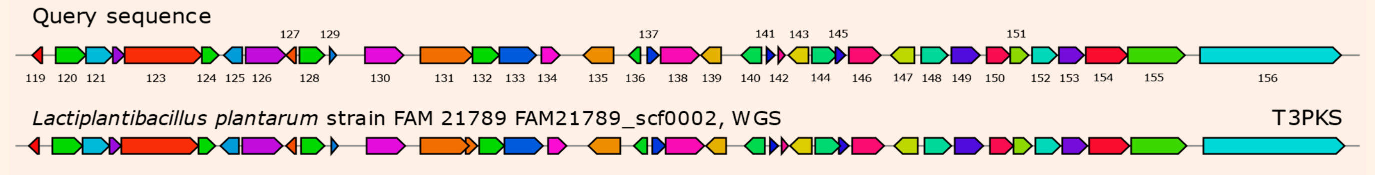

Table S2. (continued) Identified secondary metabolic regions in *Lp. plantarum* strains with antiSMASH and their corresponding ClusterBLAST scores. Significant hit for NODE_3 linked to NZ_VBSY01000002 *Lactiplantibacillus plantarum* strain FAM 21789 FAM21789_scf0002, whole genome shotgun sequence.

(B) KK1 strain: NODE_3

**cluster type**	**T3PKS** (Type III PolyKetide Synthase)						
**gene (locus tag)**	**locus**			**annotation**			
	**start (nt)**	**finish (nt)**	**strand**	**% identity**	**blast score**	**% coverage**	**e-value**
ctg3_119	128868	129153	–	100	188	100.0	$5.93e^{-62}$
ctg3_120	129585	130521	+	100	629	100.0	$2.23e^{-229}$
ctg3_121	130530	131322	+	100	501	100.0	$2.21e^{-180}$
ctg3_122	131334	131685	+	100	203	100.0	$3.32e^{-67}$
ctg3_123	131706	134043	+	100	1492	100.0	0.0
ctg3_124	134061	134580	+	100	343	100.0	$4.74e^{-121}$
ctg3_125	134722	135298	–	100	369	100.0	$2.47e^{-130}$
ctg3_126	135413	136625	+	100	735	100.0	$2.45e^{-268}$
ctg3_127	136642	136930	–	99	160	86.3	$1.14e^{-50}$
ctg3_128	137041	137803	+	98	504	100.0	$8.76e^{-182}$
ctg3_129	137978	138185	+	100	140	100.0	$7.78e^{-44}$
ctg3_130	139050	140262	+	99	798	100.0	$3.53e^{-293}$
ctg3_131	140740	142345	+	100	254	22.7	$4.79e^{-81}$
ctg3_132	142363	143155	+	100	510	100.0	$4.86e^{-184}$
ctg3_133	143159	144320	+	100	766	100.0	$5.95e^{-281}$
ctg3_134	144471	145044	+	99	367	100.0	$6.55e^{-130}$
ctg3_135	145743	146685	–	100	620	100.0	$5.89e^{-226}$
ctg3_136	147130	147523	–	97	249	100.0	$6.62e^{-85}$
ctg3_137	147686	148079	+	99	283	100.0	$1.23e^{-98}$
ctg3_138	148114	149284	+	97	743	100.0	$7.4e^{-272}$
ctg3_139	149333	149963	–	97	340	100.0	$1.29e^{-118}$
ctg3_140	150586	151219	–	100	416	100.0	$1.85e^{-148}$
ctg3_141	151337	151610	+	99	143	87.8	$1.6e^{-44}$
ctg3_142	151713	151944	+	99	143	100.0	$8.7e^{-45}$
ctg3_143	152000	152636	–	99	422	100.0	$5.12e^{-151}$
ctg3_144	152747	153506	+	100	492	100.0	$3.04e^{-177}$
ctg3_145	153489	153795	+	100	197	100.0	$2.15e^{-65}$
ctg3_146	153879	154878	+	100	654	100.0	$2.01e^{-238}$
ctg3_147	155167	155890	–	100	480	100.0	$9.11e^{-173}$
ctg3_148	156114	156918	+	100	514	100.0	$1.98e^{-185}$
ctg3_149	157020	157899	+	100	538	100.0	$6.85e^{-194}$
ctg3_150	158098	158821	+	100	459	100.0	$1.1e^{-164}$
ctg3_151	158822	159386	+	100	319	100.0	$5.93e^{-111}$
ctg3_152	159505	160285	+	100	527	100.0	$6.97e^{-191}$
ctg3_153	160300	161086	+	100	493	100.0	$3.01e^{-177}$
ctg3_154	161123	162401	+	100	802	100.0	$5.9e^{-294}$
ctg3_155	162440	164150	+	100	1117	100.0	0.0
ctg3_156	164643	168957	+	100	2860	100.0	0.0
Number of proteins with BLAST hits to this cluster: **38**							
Cumulative BLAST score: **21546.0**							

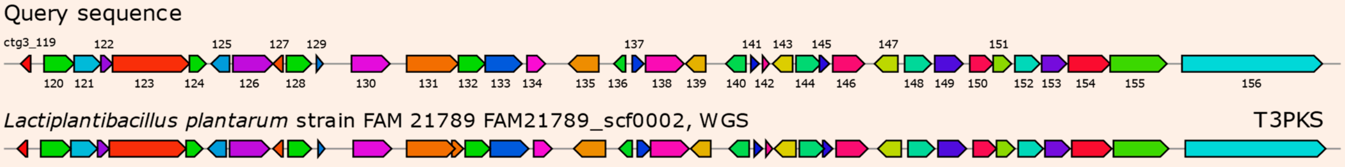

Table S3. epsSMASH - ClusterBLAST scores for *Lp. plantarum* strain DM1 on:

(A) NODE 3

**significant hit**	NZ_BCMI01000030<br>*Secundilactobacillus pentosiphilus* strain IWT25, whole genome shotgun sequence						
**cluster class**	putative wzy-dependent pathway no OPX (outer membrane porin) gene						
**cluster type**	*Lactobacillus* EPS (exopolysaccharides)						
**gene**	**locus**			**annotation**			
**(locus tag)**	**start (nt)**	**finish (nt)**	**strand**	**% identity**	**blast score**	**% coverage**	**e-value**
ctg3_104	110598	111357	+	35	122	102.4	6.81e^{-33}
ctg3_105	111374	112082	+	46	189	91.1	2.16e^{-59}
ctg3_106	112020	112842	+	45	212	93.0	9.62e^{-68}
ctg3_107	112857	113799	+	54	324	97.4	2.22e^{-110}
ctg3_108	113785	114451	+	63	291	95.9	2.3e^{-100}
ctg3_112	117843	118812	+	35	99	61.2	2.46e^{-23}
Number of proteins with BLAST hits to this cluster: **6**							
Cumulative BLAST score: **1237.0**							

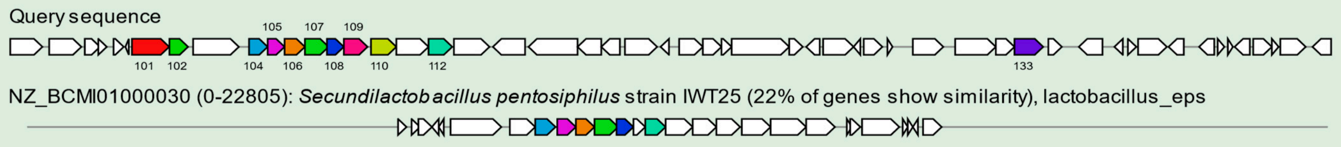

(B) NODE 29

**significant hit**	NZ_JAHAVQ010000016<br>*Liquorilactobacillus sicerae* strain, whole genome shotgun sequence						
**cluster class**	alpha-glucansucrase (sucrase-dependent pathway)						
**cluster type**	*Lactobacillus* EPS (exopolysaccharides)						
**gene**	**locus**			**annotation**			
**(locus tag)**	**start (nt)**	**finish (nt)**	**strand**	**% identity**	**blast score**	**% coverage**	**e-value**
ctg29_2	2890	4129	–	45	345	99.76	1.41e^{-115}
ctg29_4	5712	6642	–	92	207	33.98	1.54e^{-67}
Number of proteins with BLAST hits to this cluster: **2**							
Cumulative BLAST score: **552.0**							

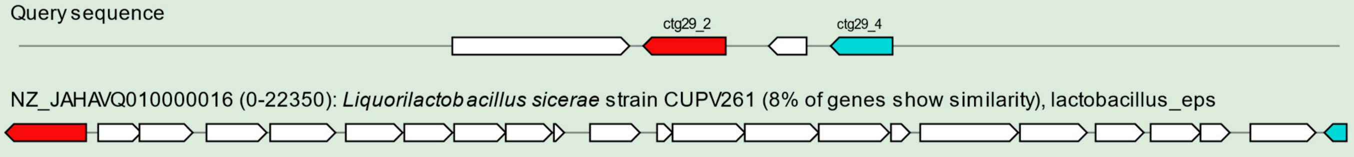

Table S3. (continued) epsSMASH - ClusterBLAST scores for *Lp. plantarum* strain DM1 on:

(C) NODE 37

**significant hit**	NZ_AYYY01000055 *Paucilactobacillus vaccinostercus* DSM 20634 NODE_105, whole genome shotgun sequence						
**cluster class**	putative wzy-dependent pathway no OPX (outer membrane porin) gene						
**cluster type**	*Lactobacillus* EPS (exopolysaccharides)						
**gene**	**locus**			**annotation**			
**(locus tag)**	**start (nt)**	**finish (nt)**	**strand**	**% identity**	**blast score**	**% coverage**	**e-value**
ctg37_2	1264	2758	–	43	387	99.8	$6.5e^{-130}$
ctg37_5	5062	6052	–	34	116	65.7	$1.84e^{-29}$
ctg37_7	6715	7687	–	31	104	79.6	$3.94e^{-25}$
ctg37_8	8294	9089	–	45	234	96.97	$8.56e^{-77}$
ctg37_9	9054	9783	–	53	210	87.2	$1.1e^{-67}$
ctg37_10	9794	10562	–	36	133	101.6	$4.18e^{-37}$
Number of proteins with BLAST hits to this cluster: **6**							
Cumulative BLAST score: **1184.0**							

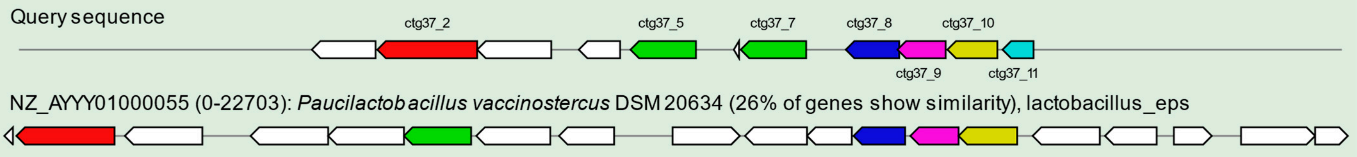

Table S4. epsSMASH - ClusterBLAST scores for *Lp. plantarum* strain KK1 on:

(A) NODE 3

**significant hit**	NZ_BCMI01000030 *Secundilactobacillus pentosiphilus* strain IWT25, whole genome shotgun sequence						
**cluster class**	putative wzy-dependent pathway no OPX (outer membrane porin) gene						
**cluster type**	*Lactobacillus* EPS (exopolysaccharides)						
**gene (locus tag)**	**locus**			**annotation**			
	**start (nt)**	**finish (nt)**	**strand**	**% identity**	**blast score**	**% coverage**	**e-value**
ctg3_104	110598	111357	+	35	122	102.4	$6.81e^{-33}$
ctg3_105	111374	112082	+	46	189	91.1	$2.16e^{-59}$
ctg3_106	112020	112842	+	45	212	93.0	$9.62e^{-68}$
ctg3_107	112857	113799	+	54	324	97.4	$2.22e^{-110}$
ctg3_108	113785	114451	+	63	291	95.9	$2.3e^{-100}$
ctg3_112	117843	118812	+	35	99	61.2	$2.46e^{-23}$
Number of proteins with BLAST hits to this cluster: **6**							
Cumulative BLAST score: **1237.0**							

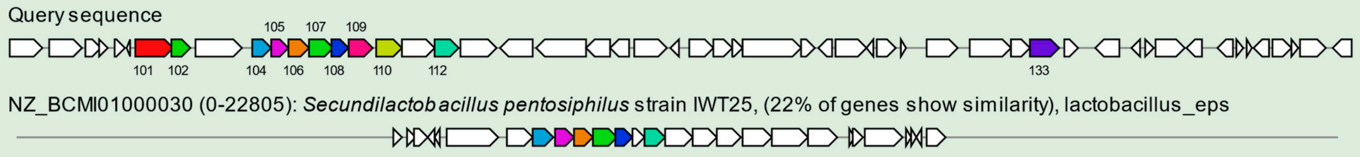

(B) NODE 34

**significant hit**	NZ_AYYY01000055 *Paucilactobacillus vaccinostercus* DSM 20634 NODE_105, whole genome shotgun sequence						
**cluster class**	putative wzy-dependent pathway no OPX (outer membrane porin) gene						
**cluster type**	*Lactobacillus* EPS (exopolysaccharides)						
**gene (locus tag)**	**locus**			**annotation**			
	**start (nt)**	**finish (nt)**	**strand**	**% identity**	**blast score**	**% coverage**	**e-value**
ctg37_2	1264	2758	–	43	387	99.8	$6.5e^{-130}$
ctg37_5	5062	6052	–	34	116	65.7	$1.84e^{-29}$
ctg37_7	6715	7687	–	31	104	79.6	$3.94e^{-25}$
ctg37_8	8294	9089	–	45	234	96.97	$8.56e^{-77}$
ctg37_9	9054	9783	–	53	210	87.2	$1.1e^{-67}$
ctg37_10	9794	10562	–	36	133	101.6	$4.18e^{-37}$
Number of proteins with BLAST hits to this cluster: **6**							
Cumulative BLAST score: **1184.0**							

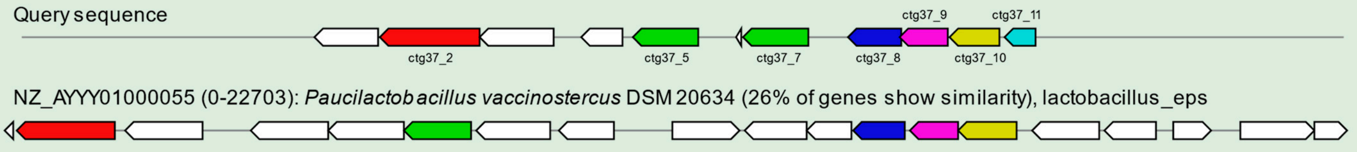

(C) NODE 42 – no significant ClusterBlast hits found.

Table S5. ClusterBLAST scores for *Lp. plantarum* strain DM1 at NODE_45 using:

(A) antiSMASH database hit

**significant hit**	NZ_CP023728 *Lactiplantibacillus plantarum* strain 10CH chromosome, complete genome						
**cluster type**	**RiPP-like** (Ribosomally synthesized and post-translationally modified Peptide Product)						
**gene**	**locus**			**annotation**			
**(locus tag)**	**start (nt)**	**finish (nt)**	**strand**	**% identity**	**blast score**	**% coverage**	**e-value**
ctg45_1	53	1703	–	99	1009	98.7	0.0
ctg45_2	1969	2134	+	61	50	103.7	$3.28e^{-08}$
ctg45_3	2158	2317	+	100	108	100.0	$1.22e^{-31}$
ctg45_4	2415	3189	+	100	468	100.0	$2.54e^{-167}$
ctg45_5	3489	4233	–	100	480	100.0	$1.1e^{-172}$
ctg45_6	4233	5031	–	46	225	98.5	$1.02e^{-69}$
Number of proteins with BLAST hits to this cluster: **6**							
Cumulative BLAST score: **2630.0**							

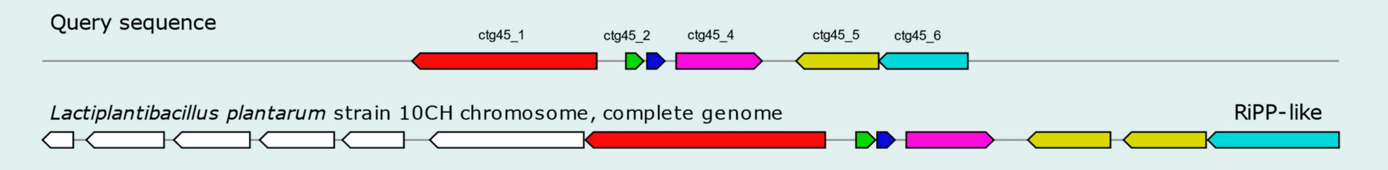

(B) BAGEL4 database hit with Plantaricin_E (Bit Score=56.9954)

**gene**	**locus**			**annotation**		
**(ORF)**	**start (nt)**	**finish (nt)**	**strand**	**% match**	**e-value**	**function**
orf00001	54	1703	–	99.45	0.0	bacteriocin ABC-transporter, ATP-binding and permease protein PlnG
orf00003	1970	2134	+	60.71	$3.31e^{-16}$	170.2; Plantaricin_E
orfblast_1	2158	2314	+			171.2; Plantaricin_F
orf00005	2196	2336	+			
orf00007	2398	3189	+	100.00	0.0	P71468_LACPL PlnI (immunity protein PlnI, membrane-bound protease CAAX family)
orf00009	3490	4161	–	99.10	$2.11e^{-161}$	response regulator PlnD
orf00010	4234	5031	–	24.00	$4.17e^{-13}$	bacteriocin production related histidine kinase

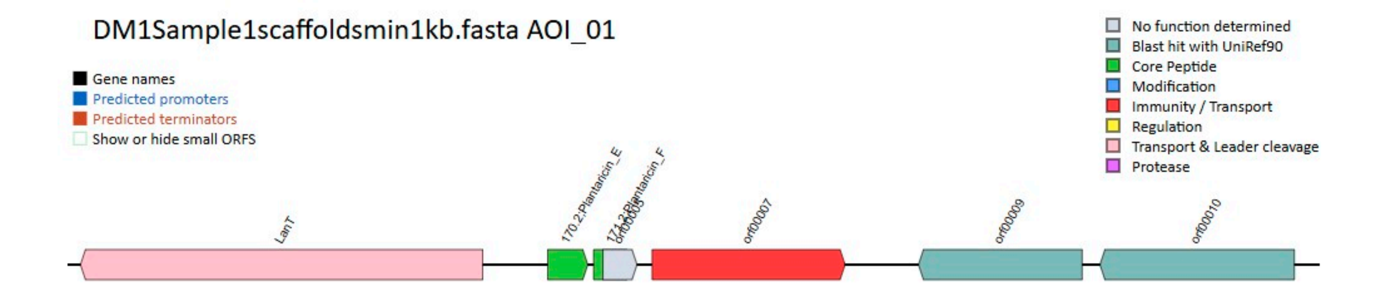

Table S6. ClusterBLAST scores for *Lp. plantarum* KK1 strain at NODE_41 using:

(A) antiSMASH database hit

**significant hit**	NC_021514<br>*Lactiplantibacillus plantarum* 16, complete sequence						
**cluster type**	**RiPP-like**<br>(Ribosomally synthesized and post-translationally modified Peptide Product)						
**gene**	**locus**			**annotation**			
**(locus tag)**	**start (nt)**	**finish (nt)**	**strand**	**% identity**	**blast score**	**% coverage**	**e-value**
ctg41_1	103	901	+	100	429	85.3	1.98e-152
ctg41_2	901	1645	+	100	482	100.0	2.7e-173
ctg41_3	1945	2719	–	100	468	100.0	2.54e-167
ctg41_4	2817	2976	–	100	108	100.0	1.22e-31
ctg41_5	3000	3165	–	100	100	100.0	2.25e-28
ctg41_6	3431	5081	+	100	1011	98.7	0.0
Number of proteins with BLAST hits to this cluster: **6**							
Cumulative BLAST score: **2598.0**							

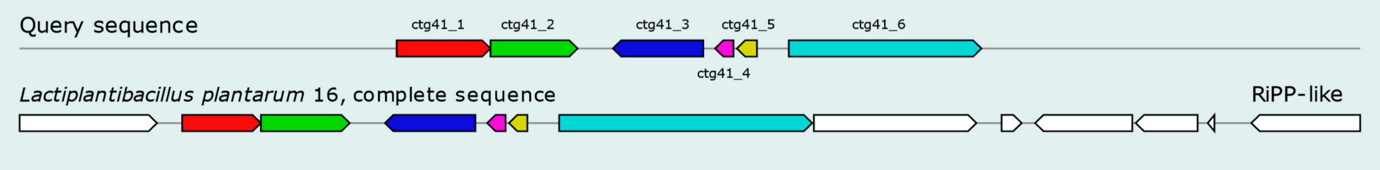

(B) BAGEL4 database hit with Plantaricin_E (Bit Score=56.995)

**gene**	**locus**			**annotation**		
**(ORF)**	**start (nt)**	**finish (nt)**	**strand**	**% match**	**e-value**	**function**
orf00001	104	901	+	24.00	4.17e-13	bacteriocin production related histidine kinase
orf00002	974	1645	+	99.10	2.11e-161	response regulator PlnD
orf00004	1946	2737	–	100.00	0.0	P71468_LACPL PlnI (immunity protein PlnI, membrane-bound protease CAAX family)
orf00006	2799	2939	–			
orfblast_1	2820	2976	–			171.2; Plantaricin_F
orf00008	3001	3165	–	60.71	3.31e-16	170.2; Plantaricin_E
orf00010	3432	5081	+	99.45	0.0	bacteriocin ABC-transporter, ATP-binding & permease PlnG

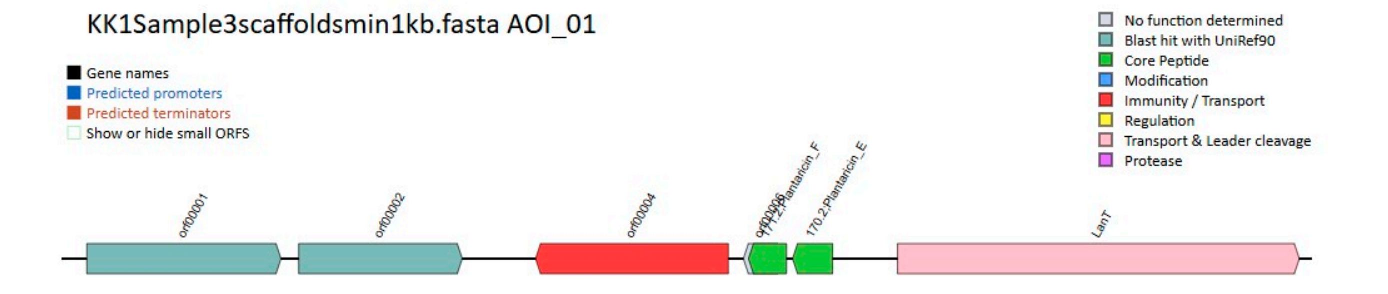

Supplement: Supplementary file 1 [file foods-15-02148-s001.zip › foods-4309864-supplementary.pdf]
